# Granuloma annulare and necrobiosis lipoidica in a patient with *HNF1A*-MODY

**DOI:** 10.20945/2359-3997000000477

**Published:** 2022-05-12

**Authors:** Gabriela Irene Garcia Brandes, Renata Peixoto-Barbosa, Ana Paula Gomes Meski, Fernando M. A. Giuffrida, André F. Reis

**Affiliations:** 1 Universidade Federal de São Paulo Departamento de Dermatologia São Paulo SP Brazil Departamento de Dermatologia, Universidade Federal de São Paulo (Unifesp), São Paulo, SP, Brasil; 2 Universidade Federal de São Paulo Disciplina de Endocrinologia São Paulo SP Brazil Disciplina de Endocrinologia, Universidade Federal de São Paulo (Unifesp), São Paulo, SP, Brasil; 3 Universidade do Estado da Bahia Departamento de Ciências da Vida Salvador BA Brazil Departamento de Ciências da Vida, Universidade do Estado da Bahia (UNEB), Salvador, BA, Brasil; 4 Universidade de São Paulo Hospital das Clínicas Departamento de Dermatologia São Paulo SP Brazil Departamento de Dermatologia, Hospital das Clínicas, Universidade de São Paulo (USP), São Paulo, SP, Brasil

## Abstract

Maturity-onset diabetes of the young (MODY) is a heterogeneous group of monogenic forms of diabetes mellitus with distinct clinical features. Clinical dermatological phenotypes in MODY patients are very rare in literature. This report describes a patient with *HNF1A*-MODY presenting with necrobiosis lipoidica (NL) and granuloma annulare (GA). A 39-year-old asymptomatic woman, with atypical diabetes diagnosed at age 17, has a confirmed *HNF1A* mutation on exon 2 (c.392G>A, p.R131Q), classified as Pathogenic by the ACMG guidelines. She has reasonable metabolic control using oral anti-diabetic medications and has no chronic diabetic complications. Clinical and histologic diagnoses of both NL and GA were made. We discuss these conditions and their association with MODY.

## INTRODUCTION

Maturity-onset diabetes of the young (MODY) is a heterogeneous group of monogenic forms of diabetes mellitus, grouped within distinct clinical syndromes. Mutations in hepatocyte nuclear factor-1 homeobox A (*HNF1A*) are among the most common causes of MODY and show marked clinical heterogeneity (1). In *HNF1A*-MODY, frequency of micro- and macrovascular chronic complications is similar to patients with type 1 diabetes (T1D) and type 2 diabetes (T2D) (2). Clinical phenotypes in patients with MODY due to mutations in different genes have been increasingly described. Nevertheless, dermatological phenotypes in MODY patients are seldom found in literature.

Dermatological manifestations are found in about one-third of all people with diabetes, reaching close to 70% in T1D (3). Necrobiosis lipoidica (NL) is a granulomatous condition presenting as indolent atrophic plaques, often in lower extremities (4). NL occurs in association with diabetes, accounting for the past use of the term “necrobiosis lipoidica diabeticorum” to describe this disease. However, the recognition that NL also occurs in the absence of diabetes led to a change in terminology (5). Granuloma annulare (GA) is an idiopathic disorder affecting the dermis and subcutaneous tissues, classically presenting as annular groups of papules ranging from skin-colored to erythematous, localized at the dorsal hands and/or feet (6). Rarely, both dermatoses have been reported to occur concomitantly in the same patient (7). There are only a few descriptions of either of these lesions in patients with MODY mutations (8,9). Reports of both lesions occurring concomitantly in an individual with MODY are even rarer (10). In this report, we describe a patient with *HNF1A*-MODY and presence of both NL and GA.

## CASE REPORT

We report the case of a 39-year-old asymptomatic woman, diagnosed with hyperglycemia during a routine visit when she was 17 years old (fasting glucose 145 mg/dL; HbA1c 6.8%). Further testing showed C-peptide 2.01 ng/mL, negative pancreatic autoantibodies (anti-GAD, anti-IA2, anti-insulin), high-sensitivity C-reactive protein (hsCRP) 0.3 mg/L, creatinine 0.8 mg/dL, and normal lipid profile. Glycosuria was detected in several samples. She was prescribed metformin and gliclazide with good metabolic control (HbA1c always below 7.2%, last 6.6%). She shows no chronic diabetic complications such as retinopathy, nephropathy, or neuropathy. She has no personal history of cardiovascular disease, with a previous coronary artery calcium (CAC) score of zero.

One year before diabetes was diagnosed, at age 16, she presented with rashes in her foot characterized by a brownish erythematous patch with papular borders and depressed center, receiving a clinical diagnosis of GA, confirmed shortly afterwards by a biopsy demonstrating preserved epidermis, areas of degenerated collagen in the dermis surrounded by a proliferation of lymphocytes and histiocytes in palisade, in addition to giant cells. She used topical corticosteroids without improvement.

Five years later, at age 21, she presented well-defined, oval, slightly erythematous plaques in her left shin, with atrophic center and regular limits, measuring 5 cm of diameter. A clinical diagnosis of NL was made. A new biopsy revealed collagen swelling with nuclear fragmentation, necrotic areas, histiocytes with epithelioid aspect, giant cells, and vessels with lymphocytic infiltrate in concentric arrangement with dense plasma cells foci, thus confirming the diagnosis of NL. She received topical corticosteroids and vitamin E without significant improvement.

**Figure 1:**
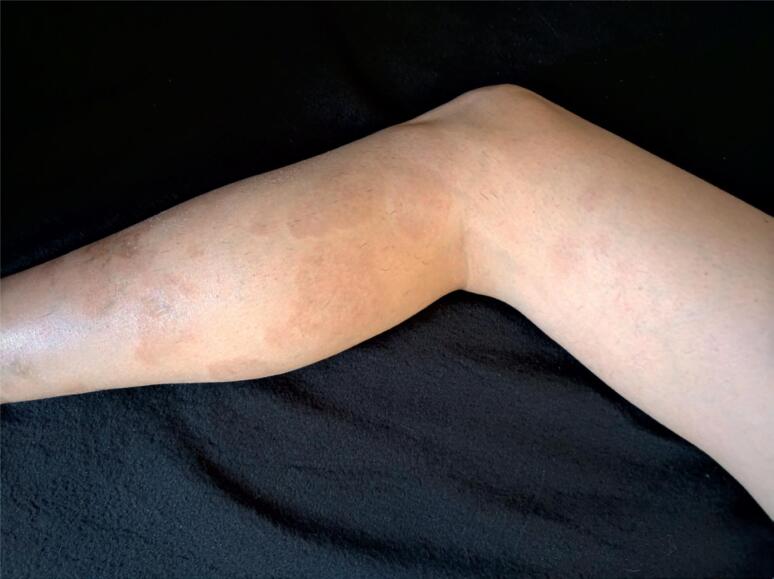
Note the GA with red brownish plaques, some with an erythematous halo, ranging from 1 to 8 cm in diameter, grouped and distributed in lower limbs.

**Figure 2:**
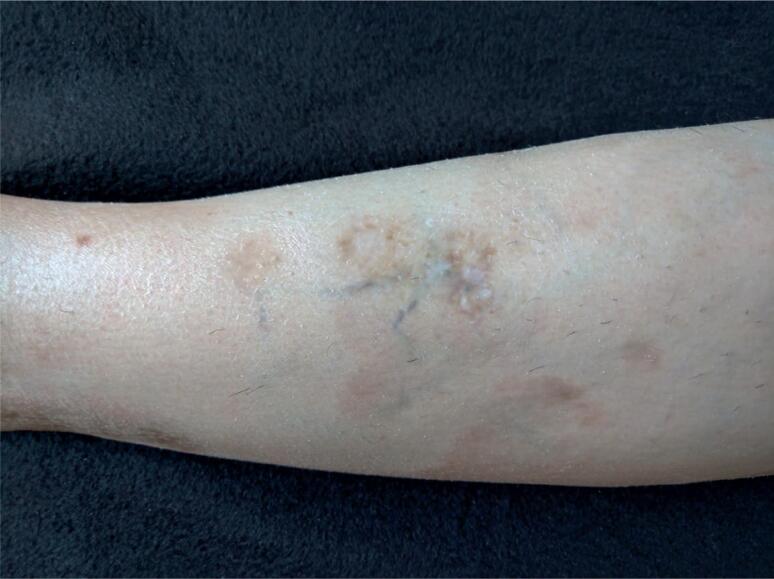
Note the plaques present in the anterior tibial area, with shiny porcelain-like atrophic center and telangiectasia typical of NL.

Current physical examination showed a BMI of 21.0 kg/m^2^, normal blood pressure (120 x 80 mmHg), and no other relevant alterations. Her current skin evaluation can be seen on Figure 1 (GA) and Figure 2 (NL).

Her father had diabetes diagnosed at age 26 (BMI 27.1 kg/m^2^ at the occasion) and has been using insulin since then. She also has an aunt with diabetes since age 21 who takes no medication. The atypical clinical presentation of hyperglycemia, combined with her family background and absence of immunologic markers specific for T1D, raised the hypothesis of monogenic diabetes – more specifically *HNF1A*-MODY. Genetic testing showed a variant in exon 2 (c. 392G>A, p.R131Q) in the *HNF1A* gene, previously reported in several populations (11) and classified as Pathogenic according to the American College of Medical Genetics and Genomics (ACMG) (12). The same mutation was found in the father, but her aunt was not available for genetic testing. Of note, there is no history of skin lesions in her relatives.

## METHODS

*HNF1A* mutations were detected by previously described methods (13). The following clinical parameters were recorded: body-mass index (BMI – weight in kg divided by square of height in meters), blood pressure (measured after five minutes resting), presence of diabetic complications (albumin excretion rate [AER] above 30 mg/24 h, neuropathy by clinical examination, and retinopathy by fundoscopy). Laboratory measurements: hemoglobin A1c (HbA1c – HPLC). CAC evaluation was performed as previously reported (14).

All clinical and laboratory data were collected for a study previously approved by the Research Ethics Committee at *Universidade Federal de São Paulo* (protocol number CAAE 32784514.4.0000.5505). The patient provided written informed consent for this report.

## DISCUSSION

We describe the association of NL and GA in a patient with *HNF1A*-MODY. Descriptions of this association are scarce in literature. Marchetti and co-authors described a 12 years-old girl with both NL and GA confirmed by biopsy. However, MODY diagnosis was made only by clinical characteristics because molecular testing was not available (10). Metabolic profile of this patient showed a more severe form of diabetes (HbA1c 11.3%), commonly seen in MODY associated with transcription factor genes such as *HNF1A*-MODY (1). So, this patient had a higher likelihood of having this MODY subtype. To our knowledge, this is the only publication describing both NL and GA in a single MODY patient so far. Ng and cols. described two Chinese patients from the same family with NL and *HNF1A*-MODY, although no reference to a diagnostic biopsy was made. Of note, both patients had severe diabetes with multiple diabetic complications (9). Stride and co-authors reviewed medical records of 178 patients from 108 families fitting into classical criteria for MODY. In case evidence of rash was recognized, further details were investigated. They found five unrelated patients presenting with rash typical of NL, but only one was confirmed by biopsy. Mutations in *HNF1A* were found in three patients. Authors suggested that if NL is found in a young nonobese diabetic patient, diagnoses of MODY, as well as T1D, should be considered, especially in the presence of familial history of diabetes (8). Moreover, in this cohort, authors found NL to be a feature of 2.8% of MODY patients, a prevalence that seemed to be lower than that seen in T1D and higher than that found in T2D (15-17). Finally, Lumb and cols., in a letter about the use of sitagliptin in a patient with *HNF1A*-MODY, cited the presence of NL in their case report, without providing further details about the lesion (18).

Prevalence of NL in patients with diabetes ranges from 0.3% to 2.3%. It is noteworthy that about two-thirds of patients with diabetes and NL have T1D (3,4,19). Although concurrent diabetes is common in patients with NL, its presence is not mandatory to suggest the diagnosis. The reported prevalence of diabetes among individuals with NL varies widely, ranging from 11 to 65%. NL may precede the diagnosis of diabetes in most of them (5,16,20). Of note, in the case reported here, NL was diagnosed after diabetes and a few years after the diagnosis of GA.

The role of metabolic control on the development and prognosis of NL is under discussion (19). Glycated hemoglobin levels do not seem to affect the appearance of lesions (21). However, a systematic review suggested that glycemic control may have a role in influencing the prognosis of NL in patients with diabetes, but authors claim there is insufficient evidence to support or refute this claim (22). This association can be more evident in T1D (19).

Concerning the pathophysiology of NL in diabetes, some evidence suggests diabetic microangiopathy to be the leading etiologic link between both conditions, mainly because organs typically affected by diabetes (such as kidney and eye) have alterations in their vasculature similar to vascular changes seen in NL (23).

In this scenario, based on this proposed pathophysiological relationship, prevalence of NL in *HNF1A*-MODY patients would be at least similar to other forms of diabetes, considering that this MODY subtype shows prevalence of micro- and macroangiopathy similar to common forms of diabetes (1). In this regard, no microangiopathy was noted in our patient nor in those described by Stride and cols. (8). In the description of the Chinese family cited above, however, patients presented with severe microangiopathy (9). Thus, based on those descriptions, no relationship between microangiopathy and dermatological lesions can be established, and no pathophysiological mechanisms were investigated. Another hypothesis is related to the deposition of immunoglobulins, C3 complement, and fibrinogen in blood vessel walls of patients with NL. Antibody-mediated vasculitis could initiate blood vessel changes and subsequent necrobiosis in NL (24). There is also the hypothesis of abnormal collagen crosslinking, which could explain basement membrane thickening in NL (25). Hammer and co-authors investigated data from 64,133 patients with T1D, both with NL (n = 161) and without. Results suggested a strong correlation of NL with both metabolic control and diabetes duration in these patients. Authors also found that smoking, insulin dosage, and female gender are also associated with NL. Finally, this study demonstrated the prevalence of celiac disease to be significantly higher in patients with T1D and NL, indicating that immunological alterations may play a role in this dermatological disease (19).

From a clinical point of view, NL lesions typically present as asymptomatic, well-circumscribed papules and nodules, with active erythematous borders that slowly coalesce into plaques. Plaques appear violaceous and contain a central area that initially appears red-brown, but later progresses to yellow-brown discoloration. The central area often contains atrophic skin with telangiectasis. Lesions are generally localized bilaterally in the lower extremities, although they can occur on the face, scalp, trunk, groin, and upper extremities. Painful ulceration occurs in approximately 15% of cases (26).

GA is a common dermatological disorder affecting the dermis and subcutaneous tissues, presenting with generalized or localized lesions. GA lesions classically consist of small papules, with color varying from pale pink to erythematous or purple, arranged in coin-sized rings or in patches up to 5 cm in diameter (27). Generalized GA (GGA) is defined by the simultaneous presence of at least ten skin lesions or by widespread annular plaques and occurs in about 8%-15% of patients with GA (28). This condition is generally asymptomatic, although there may be some pruritus or even pain.

Etiology of GA remains unknown, but it has been linked to systemic illnesses, such as thyroid disease and cancer (6,7). Association of GA with diabetes is controversial and has not been clearly established (21,29), but about 12% of patients with GA have diabetes (30).

There are few descriptions of patients with both NL and GA (7,10). GA is usually transient, most cases resolving within two years, whereas NL usually follows a chronic course and may ulcerate and scar (27). Of note, in our patient, both GA and NL have a chronic presentation. Histologically, both GA and NL demonstrated focal degeneration of collagen and dermal granulomatous infiltrate (31). The epidermis is usually normal in both conditions. Granulomatous infiltration involves the whole dermis and often extends into subcutaneous fat, causing septal panniculitis. Changes consist in palisading granulomas, with histiocytes surrounding areas of degenerated collagen (4).

Traditional therapies for NL include topical and intralesional steroids, immunosuppressants, platelet inhibitors, and phototherapy, among others (26). The common practice of starting treatment with topical or intralesional corticosteroid therapy can be recommended. No clear recommendation exists for second-line therapy in case topical or intralesional corticosteroids fail (32). Treatment of GA also includes topical glucocorticoids, systemic glucocorticoids, and photochemotherapy (PUVA) (33). Vitamin E and corticosteroids used by the present patient did not result in improvement of either lesion.

In conclusion, we describe the rare association of *HNF1A*-MODY with NL and GA, skin conditions potentially chronic and challenging to treat. Studies with a greater number of patients with both confirmed molecular diagnosis of MODY and biopsy of the lesions are required, since they may help to establish this relationship and possible pathophysiological mechanisms involved.
